# Investigation of serum proteome homeostasis during radiation therapy by a quantitative proteomics approach

**DOI:** 10.1042/BSR20182319

**Published:** 2019-07-29

**Authors:** Amira Ouerhani, Giovanni Chiappetta, Oussema Souiai, Halima Mahjoubi, Joelle Vinh

**Affiliations:** 1 Tunis El Manar University, High Institute of Medical Technologies of Tunis, Laboratory of Biophysics and Medical Technologies, 9th Dr. ZouhairEssafi Street, Tunis 1006, Tunisia; 2 Laboratory of Proteomics and Biological Mass Spectrometry, USR 3149 CNRS - ESPCI Paris, PSL University, 10 rue Vauquelin, Paris cedex 05 75231, France; 3 Laboratory of BioInformatics, bioMathematics and bioStatistics (BIMS) Institute Pasteur of Tunis, Tunis, Tunisia

**Keywords:** proteomics, Radiotherapy, skin lesions

## Abstract

The purpose of the present study is to analyze the serum proteome of patients receiving Radiation Therapy (RT) at different stages of their treatment to discovery candidate biomarkers of the radiation-induced skin lesions and the molecular pathways underlying the radiation signatures. Six stages of RT treatment were monitored from patients treated because of brain cancer: before starting the treatment, during the treatment (four time points), and at 4 weeks from the last RT dose. Serum samples were analyzed by a proteomics approach based on the Data Independent Acquisition (DIA) mass spectrometry (MS). RT induced clear changes in the expression levels of 36 serum proteins. Among these, 25 proteins were down- or up-regulated significantly before the emergence of skin lesions. Some of these were still deregulated after the completion of the treatment. Few days before the appearance of the skin lesions, the levels of some proteins involved in the wound healing processes were down-regulated. The pathway analysis indicated that after partial body irradiation, the expression levels of proteins functionally involved in the acute inflammatory and immune response, lipoprotein process and blood coagulation, were deregulated.

## Introduction

Radiation therapy (RT) is the most common curative modality in different types of cancer treatments [1]. However, the ideal goal of RT is rarely completely fulfilled. Indeed, patients develop symptoms associated with severe toxicity of normal tissues, exhibiting early and/or late effects [2]. Radiation skin injury is the most common side effect of radiotherapy and radiological accidents [3]. The resultant injuries ranged in severity from erythema to necrosis [4,5] depending on many factors such as the total dose received, the volume of the treated tissue, and individual radio-sensitivity. It is critically important to understand the mechanisms underlying the evolution of radiation-induced skin lesions and discover early biological indicators useful to estimate the severity of the skin damage and to anticipate the occurrence of late cutaneous lesions.

Several published works have showed that serum proteome could be a promising source of radiation exposure bio-indicators [6,7] since, after RT, changes in serum protein levels were observed. The effects of ionizing radiation on serum proteome have been mostly studied in animal models [8]. Few studies have been realized with human samples. For example, Widlak et al. [9] performed a shotgun proteomics analysis of serum samples from patients before the starting of RT, at the end of RT and after 1 month from the end of the RT. However, this sampling timeframe have not allowed monitoring the phase before the occurrence of skin injuries. Deperas-Kaminska et al. [10] tried to cover this RT phase by an ELISA approach monitoring a reduced number of proteins. Thus, an unbiased proteomics analysis of the serum samples from RT patients covering the phase before the incoming of the skin lesions is still missing.

Liquid chromatography coupled to tandem mass spectrometry (LC-MS/MS) analysis of serum protein samples is an analytical challenge. The high dynamic range of concentrations usually precludes the identification and quantification of the lower abundance proteins.

This is particularly true when performing MS acquisitions using the traditional data-dependent mode (DDA). Indeed, the stochastic features of the precursor ion selection promotes the identification of the most abundant species. Recently, to overcome the limited dynamic range of the DDA analyses, acquisition methods in Data Independent Acquisition mode (DIA) were successfully induced [11].

In this work, we performed a comparative proteomics analysis based on a DIA-MS strategy to highlight the state of the serum proteome before the clinical detection of the skin lesions.

## Experimental

### Characteristics of patient group

Seven patients with a brain cancer participated at the present study (four males and three females). Cancers were in either the hypophysis or in the central nervous system. Three patients were diagnosed with benign hypophyseal adenoma and they were treated with postoperative RT with daily fractions of 1.8 Gy, according to the conventional five times weekly irradiation protocol, until a total dose of 54 Gy. Two patients were diagnosed with glioblastome (Grade IV, Grade II), these primary brain tumors were localized in the frontal lobe of the brain. After surgery, these patients were irradiated with daily fractions of 2 Gy, five times per week until a total dose of 60 Gy. One patient was diagnosed with low-grade astrocytoma of the inferior frontal lobe. He/She was treated with postoperative RT with daily fractions of 1.8 Gy, five times weekly for 6 weeks. One patient was diagnosed with intrasellar craniopharyngioma (Grade I) and was treated with postoperative RT with daily fractions of 1.8 Gy, five times weekly for 6 weeks. All the patients were irradiated with 6 MeV photons according three-dimensional conformal external beam radiotherapy with a linear accelerator, in the Radiotherapy Department of Salah Azaiez Institute, Tunis, Tunisia.

Similar intensities of skin lesions were observed in the analyzed group of patients. High intensity cutaneous erythema developed when patients received approximately 25–30 Gy of cumulative dose (at the end of the third week of treatment). No skin lesions were obseved during the first 2 weeks of radiotherapy. Most of the lesions were healed within 2–4 weeks after the end of RT. These toxicities were evaluated in all the patients by the members of the medical staff weekly during radiotherapy until complete healing.

The present study was reviewed and approved by the Ethics Review Board of the Salah Azaiez Institute (Tunis, Tunisia), and all patients provided written informed consent.

### Serum sample collection

Six consecutive blood samples (5 ml) were obtained by venipuncture from each patient: sample A (pre-treatment: before the start of RT); sample B (during treatment: 1 week after the start of RT); sample C (during treatment: 2 weeks after the start of RT); sample D (during treatment: 3 weeks after the start of RT); sample E (at the end of RT) and sample F (post-treatment sample: 1 month after the end of RT).

All the samples were left for 30 min at room temperature to allow clotting and then centrifuged at 1000×***g*** for 10 min at 4°C and the supernatants were collected. Each serum sample was stored at −80°C in 150 μl aliquots.

Serum protein concentrations were determined by Bradford protein assay.

### Serum sample preparation and LC-MS/MS analysis

In the first step of analysis, all the 42 serum samples were diluted 1:10 in MilliQ water and Albumin depleted using a removal kit (Pierce™ Albumin Depletion Kit, Thermo Fisher Scientific) according to the manufacturer’s protocol.

Dithiothreitol (DTT, 20 mM, Sigma–Aldrich) was added to depleted samples for 2 h at 37°C, then iodoacetamide (IAM, 55 mM, Sigma–Aldrich) was added for 30 min in the dark at room temperature, to reduce and alkylate cysteine residues. The samples were transferred in ultrafiltration devices (10 kDa cut-off, Microcon, Millipore-Merck,) to remove the excess reagents. Then protein mixtures were digested by trypsin (Roche, 1 μg per sample) overnight at 37°C with gentle shaking following the Filter Aided Sample Preparation (FASP) method [12]. The proteolysis was quenched with trifluoroacetic acid (TFA, Pierce, 1%). Capillary liquid chromatography (nanoLC) was performed with an Ultimate 3000 RSLC (Thermo Fisher Scientific). Each sample was injected (2 μl) on a reverse phase trap column (C18 Acclaim PepMap100, 5 μm, 300 μm i.d. × 5 mm) for pre-concentration and desalting. Analytical separation was realized with a reverse phase column (C18 Acclaim PepMap100, 3 μm, 75 μm i.d. × 50 cm) at constant flow rate of 300 nl/min, with a gradient of 2–50% of Buffer B in Buffer A over 120 min (Buffer A: 98:2 (v/v) water/acetonitrile (ACN, LC-MS grade Fisher Chemical, ThermoFisher Scientific), 0.1% formic acid; Buffer B: 10:90 (v/v) water/ACN, 0.1% formic acid). The LC was coupled to a Q-Exactive mass spectrometer (Thermo Fisher Scientific) equipped with a nano-electrospray ion source. DDA MS experiments consisted of one MS survey scan in the Orbitrap cell (resolution of 70000, scan range 400–2000 m/z, AGC target 3e6, maximum injection fill time 100 ms) followed by the MS/MS scans of the ten most intense precursors in HCD mode (dynamic exclusion 30 s, no singly charged precursors, resolution 17500, normalized collision energy 30, AGC target 1e5, maximum injection time 120 ms, isolation window 2 m/z).

To create a spectral library for the DIA-MS experiments, 1 μl of each sample was collected and pooled with others obtaining a mixed sample combining the features of all the patients at all the time points. The mixed sample was pre-fractioned at protein level by off-gel iso-electrofocusing (IEF) (Agilent Technologies 3100 Offgel Fractionator, 64 kVh, 4500 V, 50 μA, 200 mW) using 12 cm IPG strip pH range 3–10. Twelve fractions were collected and filtered with an ultra-filtration device (Microcon 10 kDa) to remove IEF’s buffers and to perform protein digestions (1 μg trypsin, 37°C, overnight) following the FASP method. The 12 peptide fractions were spiked with a mixture of retention time calibration peptides (Pierce Retention Time Calibration Mixture) before the nano LC-MS/MS analysis using a QExactive HF (Thermo Fisher Scientific) mass spectrometer in DDA mode. MS experiments consisted of one MS survey scan (400–2000 m/z; resolution 70000) followed by the MS/MS scans of the 20 most intense precursors (dynamic exclusion of 30 s, AGC target for MS was set at 3e6, maximum injection time of 100 ms) in HCD mode (isolation window of 2 m/z., AGC target 3e6, maximum injection time 120 ms, resolution 17500). Then each sample was spiked with the retention time calibration peptides and analyzed by LC-MS/MS in DIA mode with the Q-Exactive HF mass spectrometer under the same nanoLC conditions. The method consisted in a cycle of 40 MS/MS spectra with an isolation window of 15 m/z to cover the mass range 400–1000 m/z (resolution 30000, AGC target 5e5, maximum injection time set on ‘auto’). The raw spectra and their associated metafiles were deposited in the PRIDE Archive (http://www.ebi.ac.uk/pride/archive/) via the PRIDE partner repository with the dataset identifier PXD009587.

### Protein identification and quantification

For protein quantification in label-free mode, DDA data were processed with MaxQuant 1.5.3.30 software using a human SwissProt sequence database (May 2015 version). Methionine oxidation, cysteine carbamidomethylation, asparagine and glutamine deamidation were set as variable modifications. The ‘Match between runs’ option was activated (window 0.7 s). Protein quantification was performed using at least two unique peptides. The remaining settings were set to the default parameters.

For library building, DDA data of the off-gel IEF fractions obtained with Q Exactive HF were processed with Proteome Discoverer 2.1 (Thermo Fisher Scientific). Mascot and Sequest search engines were used with the same sequence database. Methionine oxidation, cysteine carbamidomethylation, asparagine and glutamine deamidation were set as variable modifications. MS tolerance of 5 ppm and MS/MS tolerance of 20 mmu were selected. The score threshold for the selection of MS/MS spectra was 20 for Mascot and 2.5 for Sequest. The resulting MSF file was loaded in the software Pinnacle (Optys Tech) to build the MS/MS spectral library.

DIA data were processed with Pinnacle. Minimum MS/MS dot product score was set at 0.6 and at least four matched fragments ions per spectrum should be present. Quantification was performed using the intensities of the two most intense peptides normalized by the LC total ion current.

### ELISA

CRISP3 levels were assayed in the 42 samples by ELISA kit (Abcam: ab213768) following the manufacturer’s protocol. Each sample was tested in duplicate.

### Statistical and pathway analyses

Differences in abundance levels of quantifiable proteins between pre- and post-RT treatment were evaluated by paired *t*-test or by Wilcoxon’s test, according to the normality of data. A fold change of 1.5 and 0.66, for up-regulation and down-regulation respectively, associated with a maximum *p*-value of 0.05 was considered as statistically significant.

The overall gene set corresponding to differentially regulated proteins was mapped to the Gene Ontology (GO slim) using DAVID 6.8. STRING (version 10.5, http://string-db.org) was used to generate protein–protein interaction network.

## Results

### Identification and quantification of serum proteome in response to radiation therapy

Shotgun proteomics is a powerful approach for protein identification and quantitation from biologic fluids. In this work, it was used to perform a comparative study of serum samples, from patients treated with RT, to find a molecular fingerprint of radiation-induced skin injuries. Patients, affected by different brain cancer types at different stages, were chosen in order to highlight specifically the effect of RT, thus minimizing the bias induced by the disease background. We first performed an exploratory analysis in DDA mode (Supplementary Table S1). Then, we increased the serum proteome quantification coverage by a DIA-based analysis (Supplementary Table S2).

**Figure 1:**
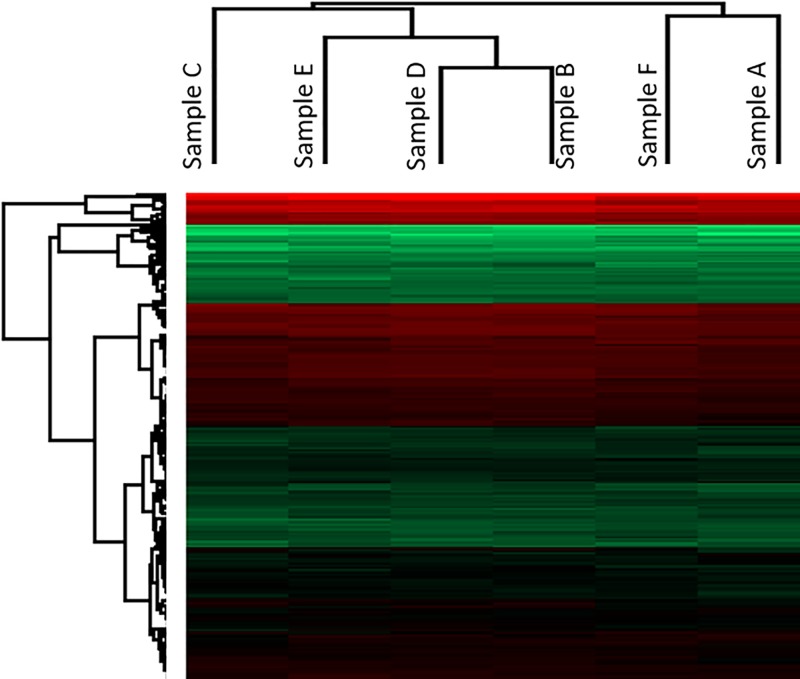
Hierarchical clustering of the serum protein samples Samples A and F show high similarity. This could be associated with the return of the serum proteome to the basal levels, after 1 month from the end of RT. Samples B, D and E show high similarity while sample C is extremely different from all the other samples.

We clustered the LC-MS/MS results considering sample similarity. The serum proteome before the start of RT and 1 month after the end of the RT are clustered together (Figure 1). Indeed, the serum proteomes returned to the basal levels 1 month after the end of RT. Samples B, D and E showed high similarity while sample C deviated from these samples. Being the last time point before the onset of the RT-associated skin burns, sample C could be interesting for diagnostic purposes.

### Early-stage treatment effects: before the emergence of skin lesions

Serum samples collected after 1 week (sample B) and 2 weeks (sample C) of RT (respectively 10 and 20 Gy of cumulated radiation) were used to monitor the early stage effects of the RT before the emergence of skin lesions. Quantitative proteomics comparison of sample A (before the start of RT) and Sample B (1 week after the start of RT) showed significant changes in 15 serum protein expression levels, 8 of which were up-regulated, while 7 were down-regulated (Table 1). Increased levels of proteins implicated in the coagulation (F5), vascularization (LRG1) and cell adhesion (JUP) correlated to the decreased levels of inhibitors of the coagulation (PROC, PLG) highlighting an initial response to the RT, to repair the tissue damages. In agreement with the data obtained with mice by Chaze et al. [8], we found decreased levels of Complement C1 protein. Interestingly we also found that the complement activator of the lectin pathway, Ficolin-3 (FCN3), was down-regulated. The up-regulation of pro-inflammatory proteins (ITH3, Haptoglobin (HP)) and the inflammatory cytoprotective proteins (Peroxiredoxin-1 and Peroxiredoxin-2 (PRDX1, PRDX2)) is also an evidence of the presence of an acute inflammatory response to the RT before the detection of any clinical manifestation of the side effects of the therapy.

**Table 1:** Proteins exhibiting significant fold changes in the early stage of the RT

Protein name	Gene	Acc. UNIPROT	MS type	*p*-value	Fold change
**Sample A vs Sample B (0 vs 10 Gy, 1st week)**					
Plasminogen	PLG	P00747	DDA	0.04	0.1
β-2-glycoprotein 1	APOH	P02749	DIA	0.02	0.2
PR domain zinc finger protein 15	PRDM15	P57071	DDA	0.04	0.3
Elongation factor 1-α 1	EEF1A1	A6NCN2	DIA	0.02	0.4
Protein C	PROC	B4DPQ7	DIA	0.02	0.4
Ficolin-3	FCN3	O75636	DIA	0.01	0.53
Junction plakoglobin	JUP	F5GWP8	DIA	0.01	1.5
Inter-α-trypsin inhibitor heavy chain 3	ITIH3	Q06033	DIA	0.001	1.58
Haptoglobin	HP	P00738	DIA	0.005	1.7
Antithrombin-III	SERPINC1	P01008	DIA	0.02	1.86
Leucine-rich α-2-glycoprotein	LRG1	P02750	DIA	0.04	2.02
Peroxiredoxin-2	PRDX2	P32119	DIA	0.04	2.47
Coagulation factor V	F5	P12259	DIA	0.04	2.7
Peroxiredoxin-1	PRDX1	Q06830	DIA	0.006	3.55
Complement C1r subcomponent	C1R	P00736	DIA/DDA	0.04/0.01	0.4/0.51
**Sample A vs Sample C (0 vs 20 Gy, 2nd week)**					
Ficolin-3	FCN3	O75636	DIA	0.01	0.46
β-2-glycoprotein 1	APOH	P02749	DIA	0.02	0.34
Plasminogen	PLG	P00747	DDA	0.04	0.17
Apolipoprotein A-II	APOA2	P02652	DIA	0.03	0.6
Actin, cytoplasmic 1	ACTB	P60709	DIA	0.02	0.32
Cysteine-rich secretory protein 3	CRISP3	P54108	DIA	0.03	0.37
Cytoplasmic FMR1 interacting protein 1	CYFIP1	E7EQ04	DIA	0.04	0.44
Fetuin-B	FETUB	Q9UGM5	DIA	0.0001	0.55
Complement factor properdin	CFP	E9PAQ1	DIA	0.04	0.41
Nucleolysin TIA-1	TIAL1	A6NKZ9	DDA	0.005	0.51
Antithrombin-III	SERPINC1	P01008	DDA	0.03	0.52
Fibronectin	FN1	P02751	DDA	0.04	0.5
Clusterin	CLU	P10909	DDA	0.04	0.58
Inter-α-trypsin inhibitor heavy chain H4	ITIH4	Q14624	DDA	0.01	1.5

Abbreviations: CFP, complement factor properdin; ITIH3, inter-α-trypsin inhibitor heavy chain H3; ITIH4, inter-α-trypsin inhibitor heavy chain H4.

The serum samples collected after 20 Gy (sample C) of radiation exhibited significant differences compared with the other time points. Sample C is the last time point before the appearance of the skin wounds (Table 1). At this point we observed mainly decreased protein levels, finding 1 up-regulated and 13 down-regulated proteins. Interestingly, we found many down-regulated proteins associated with the wound healing processes such as Fibronectin, Clusterin, Fetuin, Actin, CYFIP1, CRISP3. The decreased levels of these proteins could be a sort of breaking point leading to the appearance of the skin lesions. Similarly, to the precedent time point, the complement activator Ficolin-3 (FCN3) was decreased. Moreover, we also observed decreased levels of Complement Factor Properdin (CFP) that has similar function of FCN3, both belonging to the lectin pathway of the complment system. Compared with the previous time point, we observed that the anti-coagulation factor PLG showed similar downregulated tendency. Interestingly, the anti-coagulation factor Antithrombin III, detected up-regulated in the first week, showed decreased levels after 2 weeks (20 Gy). Platelet degranulation proteins (APOH, CLU, FN1, PLG) were found down-regulated. Taken toghether, all these observations point to an increased coagulation activity after two weeks of RT. Inflammatory processes were still detected with the increased levels of the Acute Phase Proteins (APP) inter-α-trypsin inhibitor heavy chain H4 (ITIH4) and increased PRDX2 levels (Supplementary Material S1). In contrast, we detected a return to the starting levels of the APP inter-α-trypsin inhibitor heavy chain H3 (ITIH3) and HP highlighted in Sample B.

The clustering of LC-MS/MS data showed that the sample C was different from the others. Being the last observation point before the appearance of the skin lesions, we sought to find in sample C a protein signature allowing to discriminate this clinical condition from the other therapeutic steps of the RT. Among the proteins exhibiting significant fold changes in sample C we retained Actin, CRISP3 and Ficolin-3. We tested if the concomitant detection of decreased levels of these three proteins could be a discriminatory parameter to distinguish the sample C from the others.

**Figure 2:**
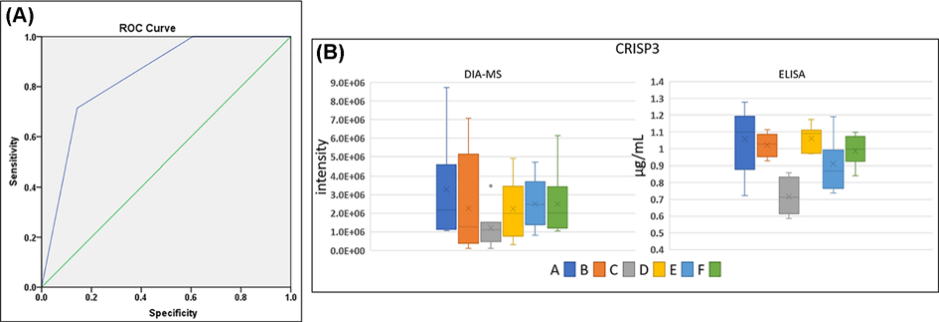
ROC curve and ELISA validation (**A**) ROC curve estimating the predictive power of Actin, CRISP3 and Ficolin-3 as candidate biomarkers of the skin burn onset. Blue line is ROC while green line is the AUC. (**B**) Box plots of CRISP3 levels measured by DIA-MS and ELISA.

The diagnostic test was considered positive if 3/3 of the selected proteins exhibited a fold change lower than 0.5 compared with sample A. Each one of the 42 samples were tested, the sensitivity and specificity of the test were evaluated by the ROC curve (Figure 2A). The resulting area under the curve (AUC) was 0.82 and a false discovery rate was 0.03 that were satisfying values to distinguish sample C from the other samples. To validate this result with an orthogonal analytical approach, the levels of the proteins CRISP3 were assessed by ELISA (Figure 2B). The tests confirmed the specific decrease in CRISP3 levels in sample C.

### Middle-stage treatment effects (when acute skin reaction reached maximal intensity, sample D and sample E)

The sample clustering showed a strict similarity between the very early treatment stage (Sample B) and the samples D and E (Figure 1), respectively, related to the 3 and 5 weeks of RT (30 and 50 Gy of cumulated radiation) (Table 2). At this stage of the therapy, patients were affected by the higher toxic effects in term of skin injuries. The similarity with Sample B was reflected by the increase in APP, ITIH3 and HP whose levels were decreased in Sample C (Supplementary Material S1). In Sample D, we detected the increase in Desmoglein-1 (DSG1), 14-3-3 protein epsilon (YWHAE) and Major histocompatibility complex, class I, B (HLA-B). DSG1 is part of the desmosome that has a key role in the wound healing in association with the Cell Junction proteins, such as JUP that was found up-regulated in Sample B [13]. However, while JUP levels returned at basal level already after 2 weeks of RT, DSG1 had higher levels from the beginning of the therapy showing its maximum at 3 weeks (Supplementary Material S1). The pro-inflammatory proteins YWHAE and HLA-B exhibited their maximum levels after 3 weeks of RT. The levels of these proteins oscillated during the therapy with a minimum at 2 weeks before the appearing of the skin lesions (Supplementary Material S1).

**Table 2:** Proteins exhibiting significant fold changes in the middle stage of the RT

Protein Name	Gene	Acc. UNIPROT	MS Type	*p*-value	Fold change
**Sample A vs Sample D (0 vs 30 Gy, 3rd week)**					
Haptoglobin	HP	P00738	DIA	0.01	1.55
Haptoglobin-related protein	HPR	P00739	DIA	0.05	1.52
Inter-α-trypsin inhibitor heavy chain 3	ITIH3	C9JX84	DIA	0.02	1.7
Peroxiredoxin-2	PRDX2	P32119	DIA	0.03	1.95
Complement factor properdin	CFP	E9PAQ1	DIA	0.04	0.41
Fibronectin	FN1	P02751	DDA	0.04	0.5
Desmoglein-1	DSG1	Q02413	DIA	0.05	3.04
14-3-3 protein epsilon	YWHAE	P62258	DIA	0.04	2.72
Major histocompatibility complex, class I, B	HLA-B	E7EQX5	DIA	0.03	1.57
**Sample A vs Sample E (0 vs 50 Gy, 5th week)**					
Complement C1r subcomponent	C1R	P00736	DDA	0.02	0.55
Inter-α-trypsin inhibitor heavy chain 3	ITIH3	C9JX84	DIA	0.007	1.56
Peroxiredoxin-2	PRDX2	P32119	DIA	0.03	1.51
β-2-glycoprotein 1	APOH	P02749	DIA	0.03	0.31
Peroxiredoxin-1	PRDX1	Q06830	DIA	0.01	4.21
Plasminogen	PLG	P00747	DDA	0.02	0.11
Nucleolysin TIA-1	TIAL1	A6NKZ9	DDA	0.01	0.54
Dermcidin	DCD	P81605	DIA	0.03	1.51
Glyceraldehyde-3-phosphate dehydrogenase	GAPDH	E7EUT4	DIA	0.02	1.73
N-acetylmuramoyl-l-alanine amidase	PGLYRP2	Q96PD5	DIA	0.04	0.56
Complement C1s subcomponent	C1S	P09871	DDA	0.02	0.59

Abbreviation: DCD, Dermicidin.

At the end of the treatment (Sample E), we detected five up-regulated proteins and six down-regulated proteins. These proteins are almost the same observed in the previous analyses. An interesting newly detected up-regulated protein is Dermicidin (DCD), a small protein (11 kDa) with antimicrobial activity thereby limiting skin infection [14].

### Post-treatment effects (1 month after the end of the treatment of RT: Sample F)

Patients after 1 month from the end of the RT showed a significant healing of the skin lesions.

**Figure 3:**
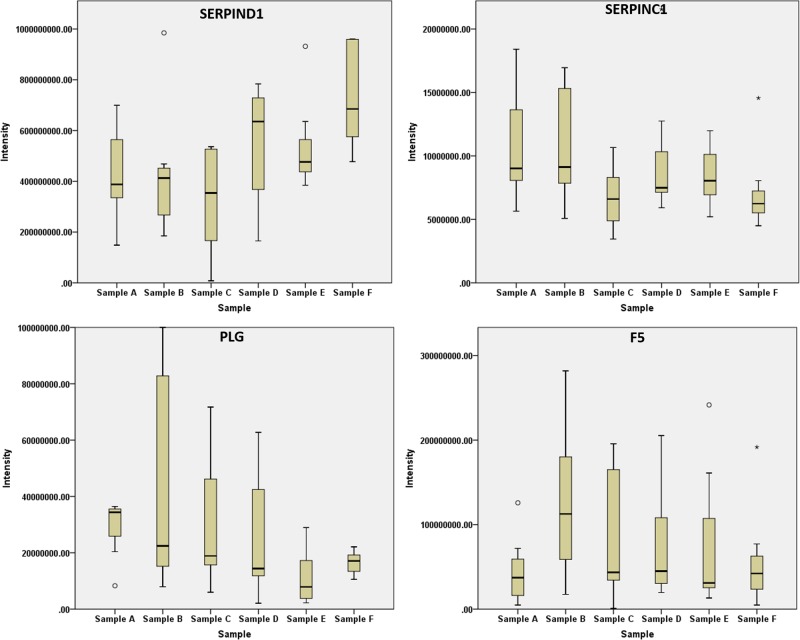
Box plots SerpinD1 shares its functions with SERPINC1 and PLG. However, with respect to the other two proteins, SERPIND1 shows a distinctive up-regulation 1 month after the end of RT. The increased levels of the anticoagulant SERPIND1 could be an indicator of the end of the healing phase considering that also the Coagulation Factor 5 (F5) returns to basal level 1 month after the end of RT.

The sample clustering showed a strict similarity between the serum protein levels of patients at 1 month after the end of the treatment and before the starting of the RT (Figure 1). Indeed, the comparison between Samples A and F evidenced very small significant differences consisting of the up-regulation of three proteins and the down-regulation of three proteins (Table 3). A newly up-regulated protein detected at this time point was the anti-coagulant Heparin cofactor (SERPIND1). SERPIND1 shares its functions with Antithrombin III (SERPINC1) and PLG. Interestingly while SEPRPINC1 and PLG showed decreased levels during the treatment with a minor increase tendency 1 month after the end, SERPIND1 showed a distinct late effect (Figure 3). In agreement with this tendency we observed that the Coagulation Factor V after an early activation returned to basal levels (Figure 3). These results support that the activation of coagulation is a very early response to RT that is gradually attenuated during the healing phase by the increase of the coagulation inhibitors. Playing an anticoagulant role could be argued that SERPIND-1 increase at this stage could be further investigated to be an indicator of the resolution of the RT-associated skin lesions. Apolipoproteins were found down-regulated at the end of RT in a previous study [9]. In agreement with this study we detected decreased levels of APOH and APOA2 in the early phase of the treatment. However, the levels of these proteins, also if not statistically significant, were decreased also after 1 month from the end of the treatment. Interestingly we found specifically increased at this point the levels of APOA1 (Figure 1 and Supplementary Material S1).

**Table 3:** Proteins exhibiting significant fold changes 1 month after the end of the RT

Protein Name	Gene	Acc. UNIPROT	MS type	*p*-value	Fold change
**Sample A vs Sample F (after 1 month from the end of RT)**					
Ficolin-3	FCN3	O75636	DIA	0.01	0.55
Peroxiredoxin-2	PRDX2	P32119	DIA	0.0006	2.05
Apolipoprotein A-I	APOA1	P02647	DIA	0.003	1.75
Fibronectin	FN1	P02751	DIA	0.01	0.39
α-2-HS-glycoprotein	AHSG	P02765	DDA	0.01	0.63
Heparin cofactor 2	SERPIND1	P05546	DIA	0.04	1.5

### Data clustering and pathway analysis

To identify potential biological pathways affected by ionizing radiation, an enrichment analysis was realized with Gene Ontology biological process (GOBP) database and Kegg’s pathways database by DAVID 6.8 [15]. The submitted dataset contained all the proteins exhibiting significant changes of expression, independently by the nature of the deregulation (36 features). Indeed, the goal of this analysis aims at defining a global map the deregulation induced by RT at serum level. We found that highly significant GOBP terms were associated with platelet degranulation (Benjamini *p*-value 7,5E-9), regulation of endopeptidase activity (Benjamini *p*-value 6,6E-4), complement activation (Benjamini *p*-value 2,9E-3), acute phase response (Benjamini *p*-value 5,1E-3), blood coagulation (Benjamini *p*-value 2,2E-2), and reverse cholesterol transport (Benjamini *p*-value 2,2E-2). Moreover, we found enriched complement and coagulation of KEGG’s pathway (Benjamini *p*-value 8,1E-7) (Supplementary Material S2).

**Figure 4:**
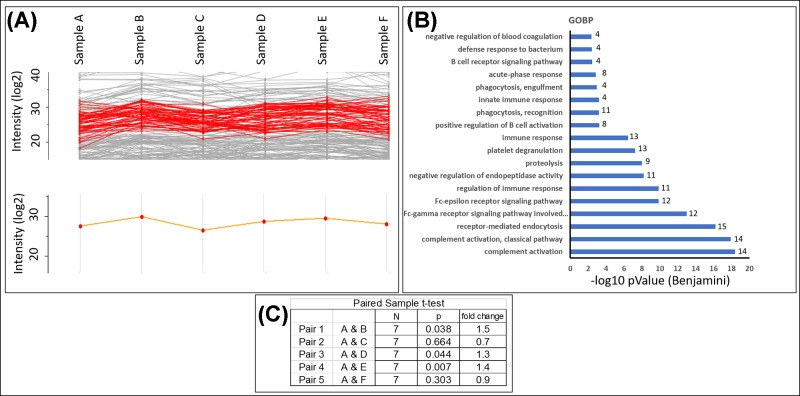
K-means clustering of the entire dataset to monitor similar protein expression profile tendencies across the time course The red curve is the cluster with the highest number of detected features (**A**). The orange curve is the average summarization of the cluster. The 55 features of the cluster were used for GO analysis and the results are reported in the histogram (**B**). The complement activation is the major biological process associated with the cluster. To evaluate if the protein level variations between time points have statistical significance, the features were summarized and five paired sample *t* tests were performed (**C**). Applying the thresholds used at protein expression level (fold change > 1.5, *p*-value <0.05 = up-regulation; fold change <0.66, *p*-value <0.05 = down-regulation), we found a significant up-regulation of the cluster at 1 week after the start of the RT.

To highlight the groups of proteins showing similar tendencies across the therapy, we performed a K-means clustering of the LC-MS/MS traces by the ‘Profile Plot’ tool in the software Perseus1.5.0.31 that allows to group by similarity measures across a time course (Figure 4A).

The most represented cluster contained 55 proteins. Submitting this dataset to DAVID 6.8 interroging the GOBP database, we found that the cluster represented the immune system process (Figure 4B). The protein abundance in this cluster oscillated during the therapy, with a minimum after 2 weeks before the appearing of the skin lesions and a maximum at the end of the radiation cycle. After 1 month from the last RT session the levels of the immune system proteins tended to return to the starting level. The highest represented subcategory of the immune system was the Classical Complement pathway with 14 features. To evaluate if the oscillation observed were statistically significant, we summarized the 14 features and we treated them as single feature in a paired sample *t*-test. It resulted that the Classical Complement pathway was up-regulated in the samples B, D, E while no differences were evidenced at 2 weeks and 1 month after the end of the treatment (Figure 4C).

**Figure 5:**
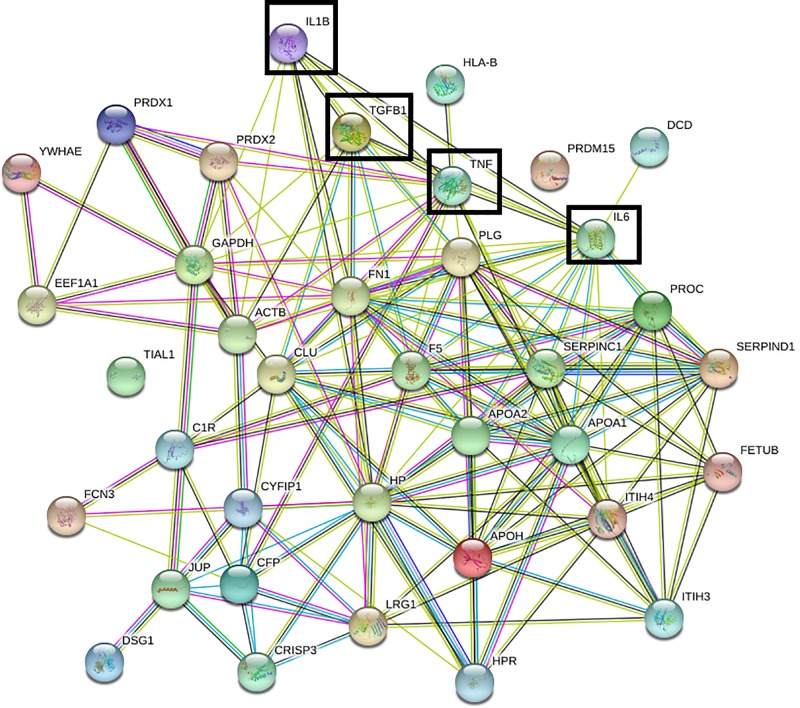
Network analysis Network of interaction obtained by STRING using the entire dataset of modulated proteins and adding the four most important biomarker of RT associated skin burns: IL-1, IL6, TNF-α and TGF-β1 (evidenced in the network by a black square).

The analysis of protein–protein interaction was performed using the list of 36 serum proteins exhibiting significant response to radiation, using STRING. We investigated the relationships between the proteins of the latter list and four proteins known as serum biomarkers of skin burns after exposure to ionizing radiation, namely IL-1β, IL6, TNF-α and TGF-β1 [16,17] (Table 4). As a result, we found an extremely interconnected subnetwork, highlighting the strong correlations between the gene set partners (Figure 5).

**Table 4:** List of the interacting proteins with the known four serum biomarkers of RT associated skin burns

Enriched subnetworks	Overlap	Overlapping proteins
Expression targets of IL6	8	GAPDH, HP, PLG, APOA1, ITIH4, SERPINC1,
		ACTB, FN1
Expression targets of TNFα	11	GAPDH, PLG, FN1, ACTB, ITIH4, HP, HLA-B,
		DSG1, SERPINC1, CLU, APOA1
Expression targets of IL-1	5	GAPDH, HP, PLG, ACTB, FN1
Expression targets of TGF-β1	10	ACTB, PLG, CLU, FN1, ITIH4, APOH, F5, GAPDH, ITIH3, YWHAE

## Discussion

In the current work, we aimed at describing the changes in the abundance levels of serum proteins from a small group of patients (seven patients) treated with radiotherapy because of brain cancer. This kind of approach was already demonstrated to be useful to highlight the side effects of the radiotherapy [9,18], such as the radiation-induced acute mucosal reaction. Acute skin lesions were observed in our patients after the accumulation of approximately 25–30 Gy who were usually healed in approximately 2–4 weeks after the end of RT. Compared with previous works [9] we added four intermediate check points for a better follow-up of the evolution of the protein levels during the RT. A similar time course study was performed in humans by Deperas-Kaminska et al. [10] using an ELISA-based approach with a preselected panel of monitored proteins. Compared with the previous studies we used a proteomics analysis based on the DIA-MS approach. As already showed by other authors, we observed that DIA-based LC-MS/MS analysis improves the protein identifications and provides more accurate quantification compared with the classic DDA approach.

The radiation therapy caused a significant increase in the levels of some proteins involved in the acute phase response especially positive APPs such as: HP, ITIH3, ITIH4 suggesting the occurrence of inflammatory processes [19]. As already demonstrated, these APPs are known to be produced in order to cure the damage induced in the skin [20] and to maintain the homeostasis [21]. Increased levels of antioxidant enzymes such as PRDX1 and PRDX2 were observed in the present study, constantly during the RT. PRDXs have been previously demonstrated as an indirect effect of RT [22] and their deregulation was detected in several reports [7,20,23,24]. PRDXs for their H_2_O_2_ scavenging activity have protective functions during inflammation events and could play the role of negative feedback of the inflammation signaling [25].

The Complement and Coagulation pathway was found enriched by bioinformatics clustering of data with the KEGG’s pathway database. We detected an early up-regulation of the pro-coagulation factor F5 and down-regulation of anticoagulant factors PLG, PROC and Antithrombin III, reflecting an activation of the coagulation system to especially repair endothelium damage [26]. F5 up-regulation is compatible with PROC down-regulation. Indeed, PROC exerts its activity by degrading F5 [27]. PROC decreased levels could be the results of the inflammatory mediators [28]. PROC levels are commonly low in sepsis. F5 is the cofactor of Factor X increasing its proteolytic activity toward Prothrombin. The level of Factor X was altered during the therapy, however the levels of its inhibitor Antithrombin III were decreased significantly at the second week and remained low also after 1 month at the end of the therapy. In agreement, Antithrombin III and Heparin systems were reportedly down-regulated during inflammatory injuries [29]. Interestingly the Heparin cofactor SERPIND1 levels increased at the end of the treatment and more significantly 1 month after the end of RT, in agreement with a decrease in inflammation and the healing of the skin injuries. PLG down-regulation is compatible with the activation of coagulation and with the activation of the Complement [30].

Although significant differences were not detectable at single protein level, we observed, by clustering the LC-MS/MS traces, that 14 proteins belonging to the Classical Complement pathway showed a similar abundance variation tendency (Figure 4). Considering these proteins as a single feature, we observed an oscillating activation of the Classical Complement pathway with a return to the basal levels at 2 weeks and at the end of the treatment. Interestingly, the activators of the Alternative and Lectin Complement pathways, respectively, Properdin and Ficolin-3 were down-regulated in the early phase of the RT. Considering these results, it should be further investigated if the selective activation of the Classical Complement pathway, associated with the repession of the Alternative and Lectin pathways, is a possible mechanism of response to the RT.

Different proteins associated with the wound healing process were down-regulated in the early phase of the RT, especially before the appearance of the skin lesions. Wound healing process is divided into three sequential phases: (i) inflammatory, (ii) proliferative and (iii) remodeling. A prolonged inflammatory phase could be detrimental to the wound healing inhibiting the evolution to the resolutive phases of the process [31]. For example, Fibronectin, an important regulator of the tissue regeneration and remodeling, is down-regulated by the inflammation [32]. Fibronectin was found down-regulated during the early phase of the treatment in our patients. Our results show at molecular level, that patients, experiencing daily irradiations, suffered from chronic inflammatory processes. These could affect the regeneration capacities of the organism until a breaking point is reached, evolving to the skin lesions. We detected this breaking point at 2 weeks of the RT when approximately 20 Gy of radiation were accumulated. Serum samples at this point showed a distinctive and widespread down-regulation of the protein levels. This tendency could be a fingerprint useful to early predict the incoming of RT-induced skin lesions. With our patients, we found that monitoring the down-regulation of Actin, CRISP3 and Ficolin-3 could allow to distinguish the phase preceding the appearance of the lesions. Obviously before to be considered a possible diagnostic marker, these observations need to be validated with a larger cohort of patients. The decreased CRISP3 levels were confirmed by ELISA. CRISP3 is an extracellular matrix protein detected in several human body fluids including saliva, sweat, blood and seminal plasma rendering it an important candidate biomarker for pathophysiological situations [33]. CRISP3 is intracellularly found in many cells of the immune system such as pre-B cells, neutrophils and eosinophils [34]. The exact function of CRISP3 remains to be established, although the occurrence of CRISP-3 in neutrophil granules and exocrine glands and its sequence homology to the pathogenesis-related proteins implicated in host defense is suggestive of a role in innate immune response [35,36]. Down-regulation of this defense molecule in our results indicate that the inactivation of CRISP3 can be an early event able to anticipate the occurrence of cutaneous lesions. Down-regulation of CRISP3 was also detected in Squamous Cell Carcinoma skin cancer [37].

Interestingly, we observed that the levels of the serum protein deregulated during the RT returned to the basal levels after 1 month from the end of RT, when the healing of the skin lesions is clinically resolved. It should be investigated if monitoring this tendency could be useful to evaluate the healing process.

## Conclusions

In this work we monitored the serum protein levels in patients undergoing RT. Compared with previous works in humans [21], we increased the sampling frequency to allow monitoring eventual peculiar changes before the occurrence of RT-induced skin lesions. We also increased the proteome coverage using a more recent analytical strategy analysis based on DIA-MS. Some key molecular factors of the wound healing process were highlighted during RT. For example, our data lead us to hypothesize the occurrence of detrimental effects on the healing process progression toward the proliferative and remodeling phases, caused by the irradiation-induced chronic inflammation.

A possible correlation between the simoultaneous decreased levels of Actin, CRISP3 and Ficolin-3 and the incoming of the skin lesions was highlighted. In perspective the predictive power of this observation should be tested on a larger cohort of patients. One month after the end of the therapy skin lesions were diagnosed mostly healed in our patients. At this point the serum proteome levels tend to return to the basal levels. In perspective, this observation could be used as an objective parameter to monitor the evolution of skin lesions healing phase during RT. In summary in this work, we evidenced some correlations between the serum proteome homestasis, the different phases of RT and its collateral effects on skin.

## Supporting information

**Supplementary Material S1:** BOX PLOTS CITED IN THE MAIN TEXT

**Supplementary Material S2:** Complement and Coagulation Cascade KEGG’s pathway

**Supplementary Table S1:** 

**Supplementary Table S2:** 

## Abbreviations

ACN: acetonitrile
AGH: Automatic Gain Control
APP: Acute Phase Protein
DDA: data-dependent mode
DIA: data independent acquisition
FASP: Filter Aided Sample Preparation
GOBP: Gene Ontology biological process
HCD: High energy Collisional Dissociation
IEF: so-electrofocusing
IPG: Immobilized pH Gradient
LC-MS/MS: liquid chromatography coupled to tandem mass spectrometry
