# Supplementary material for: Investigation of serum proteome homeostasis during radiation therapy by a quantitative proteomics approach
Source: Biosci Rep. 2019 Jul 29;39(7):BSR20182319. doi: 10.1042/BSR20182319 (PMC6663990; doi:10.1042/BSR20182319)

Investigation of serum proteome homeostasis during radiation therapy by a quantitative proteomics approach

Amira Ouerhani*[1], Giovanni Chiappetta* [2],OussemaSouiai[3], Halima Mahjoubi[1] and Joelle Vinh [2]

[1]: Tunis El Manar University, High Institute of Medical Technologies of Tunis, Laboratory of Biophysics and Medical Technologies, 9th Dr. ZouhairEssafi Street, 1006 Tunis, Tunisia;
[2]:Laboratory of Proteomics and Biological Mass Spectrometry, USR 3149 CNRS - ESPCI Paris, PSL University, 10 rueVauquelin, 75231 Paris cedex 05, France;
[3]: Laboratory of BioInformatics, bioMathematics and bioStatistics (BIMS) Institute Pasteur of Tunis, Tunisia;

.

*Authors equally contributed to this work.

Corresponding authors: giovanni.chiappetta@espci.fr, Tel +33 140 795 839

Supplementary Material S1

BOX PLOTS CITED IN THE MAIN TEXT

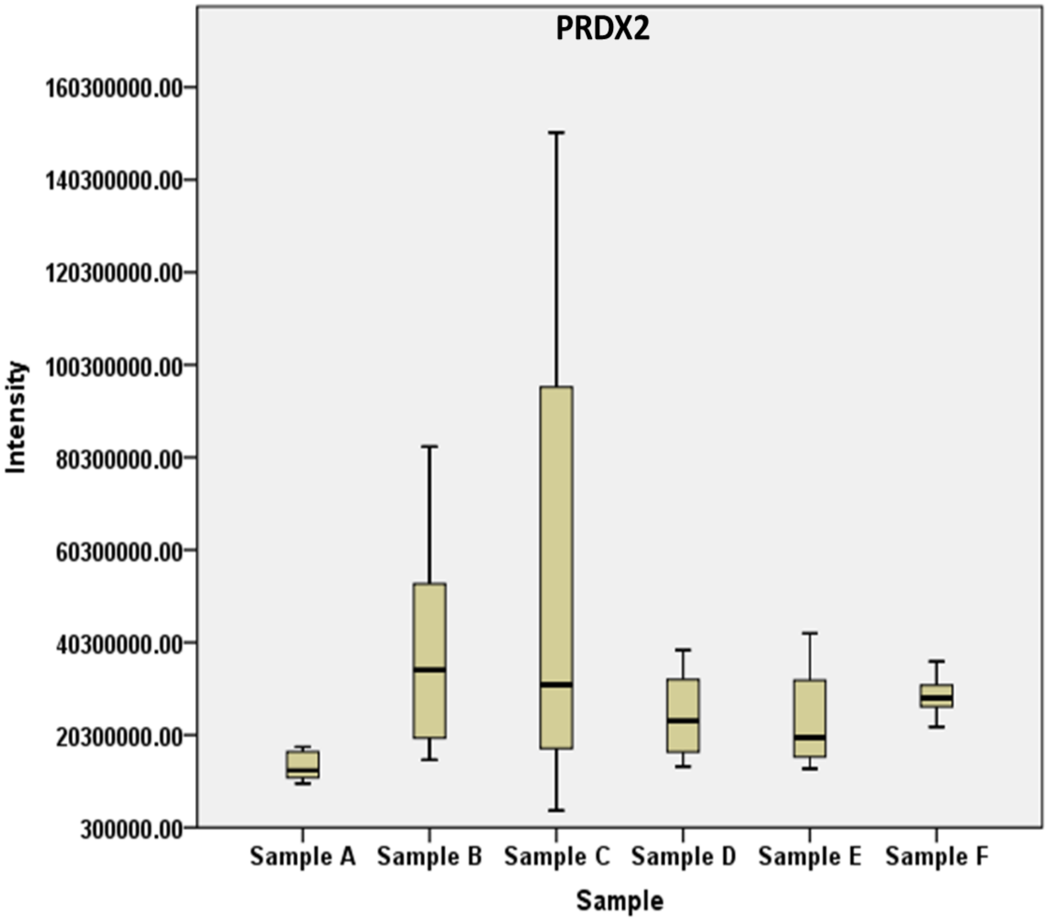

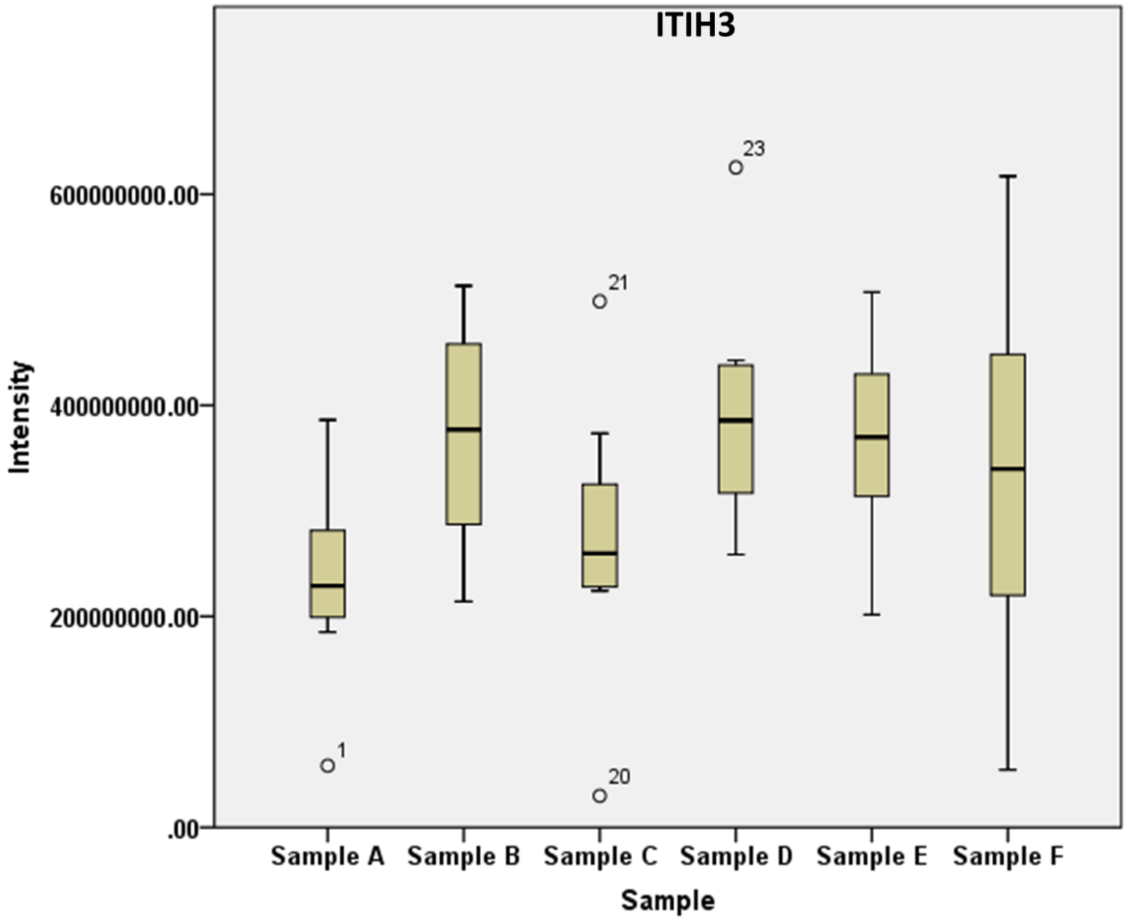
ITIH3
Intensity
600000000.00
400000000.00
200000000.00
.00
23
21
1
20
Sample A
Sample B
Sample C
Sample D
Sample E
Sample F
Sample

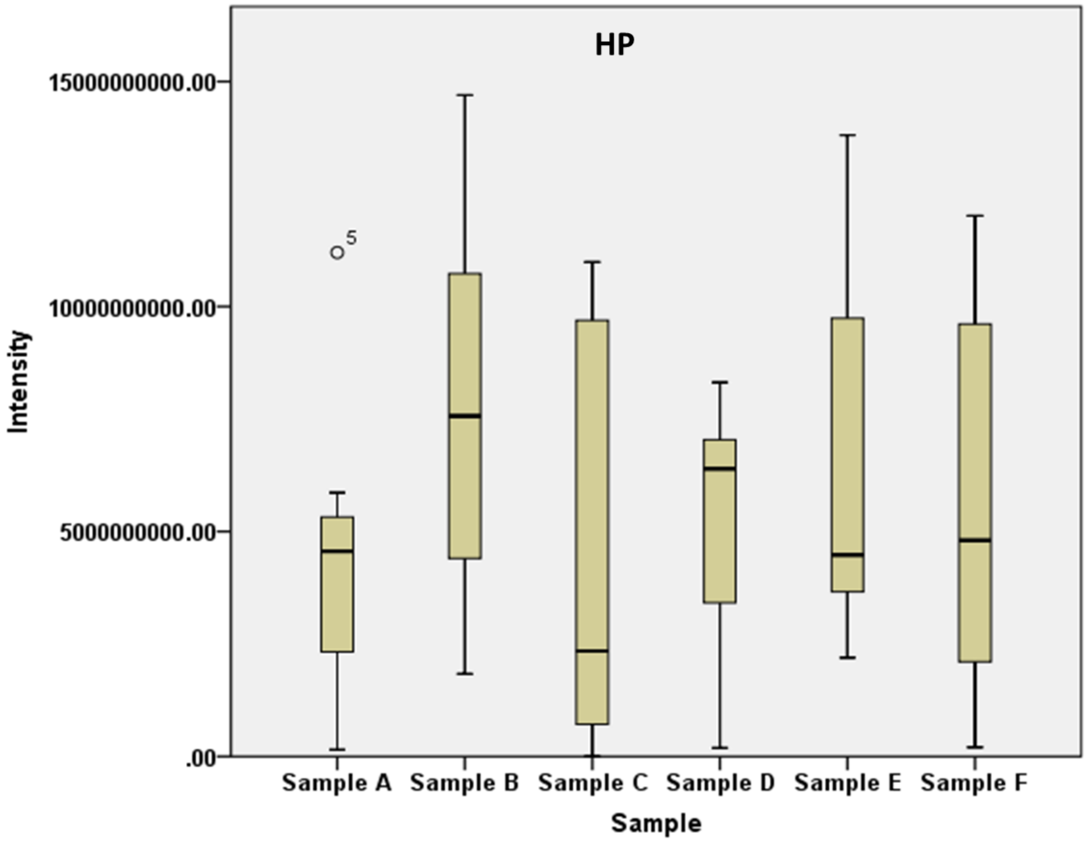

HP
15000000000.00
10000000000.00
5000000000.00
.00
Intensity
5
Sample A
Sample B
Sample C
Sample D
Sample E
Sample F
Sample

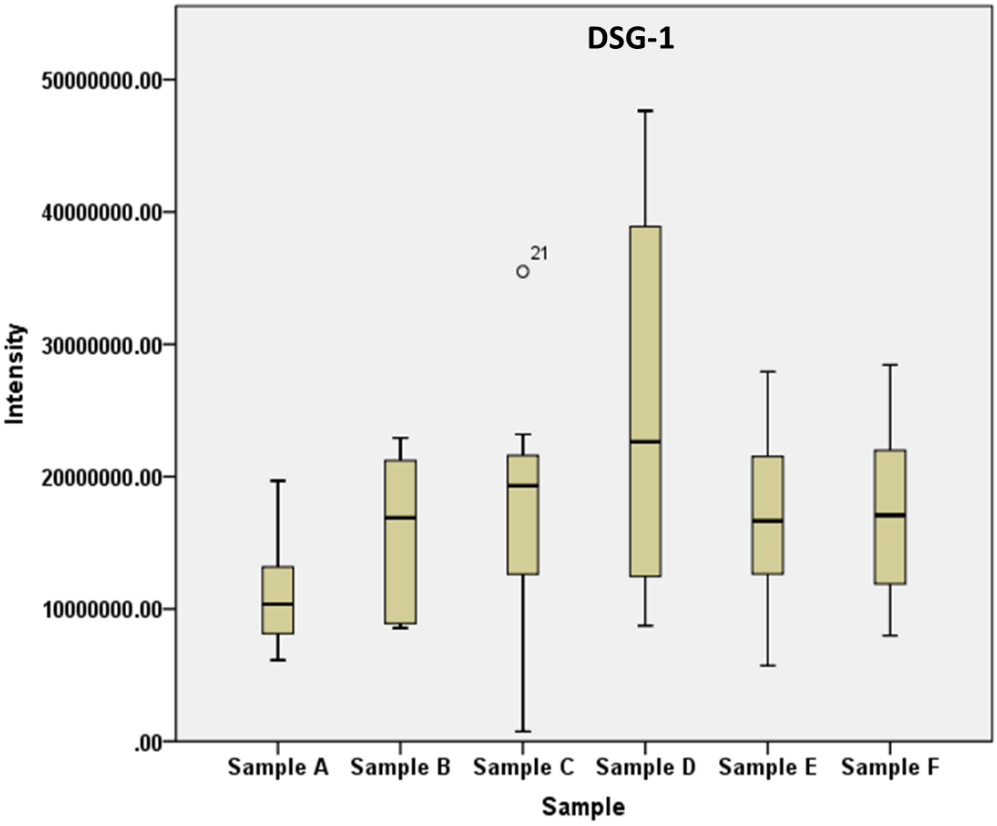
DSG-1
Intensity
50000000.00
40000000.00
30000000.00
20000000.00
10000000.00
.00
21
Sample A
Sample B
Sample C
Sample D
Sample E
Sample F
Sample

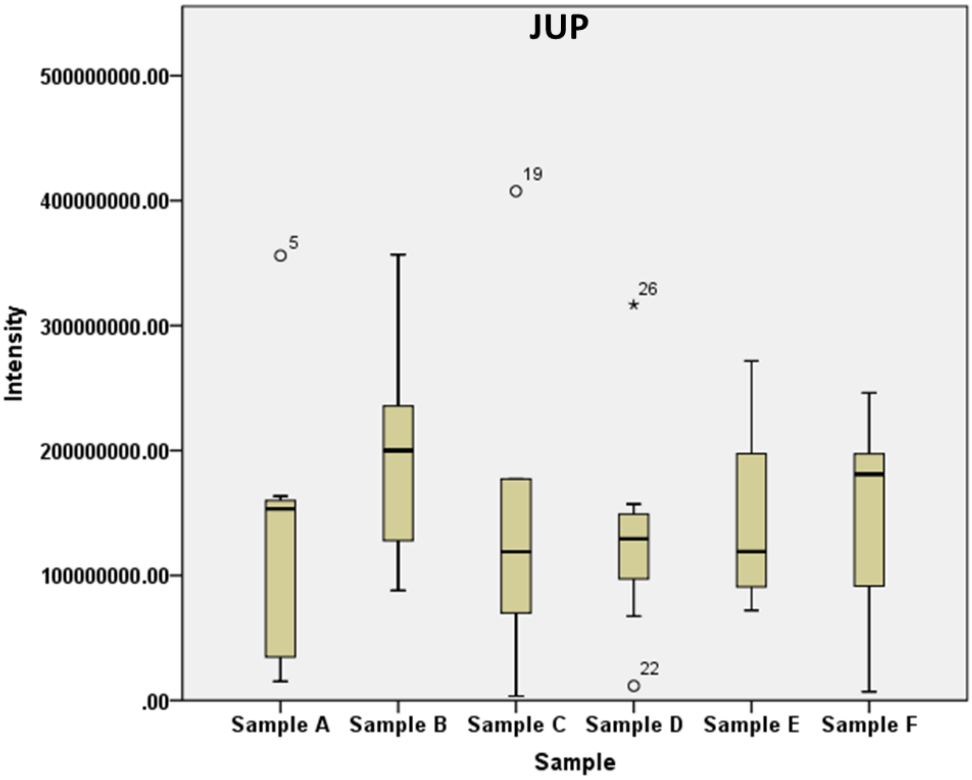
JUP
500000000.00
400000000.00
300000000.00
200000000.00
100000000.00
.00
Intensity
5
19
26
22
Sample A
Sample B
Sample C
Sample D
Sample E
Sample F
Sample

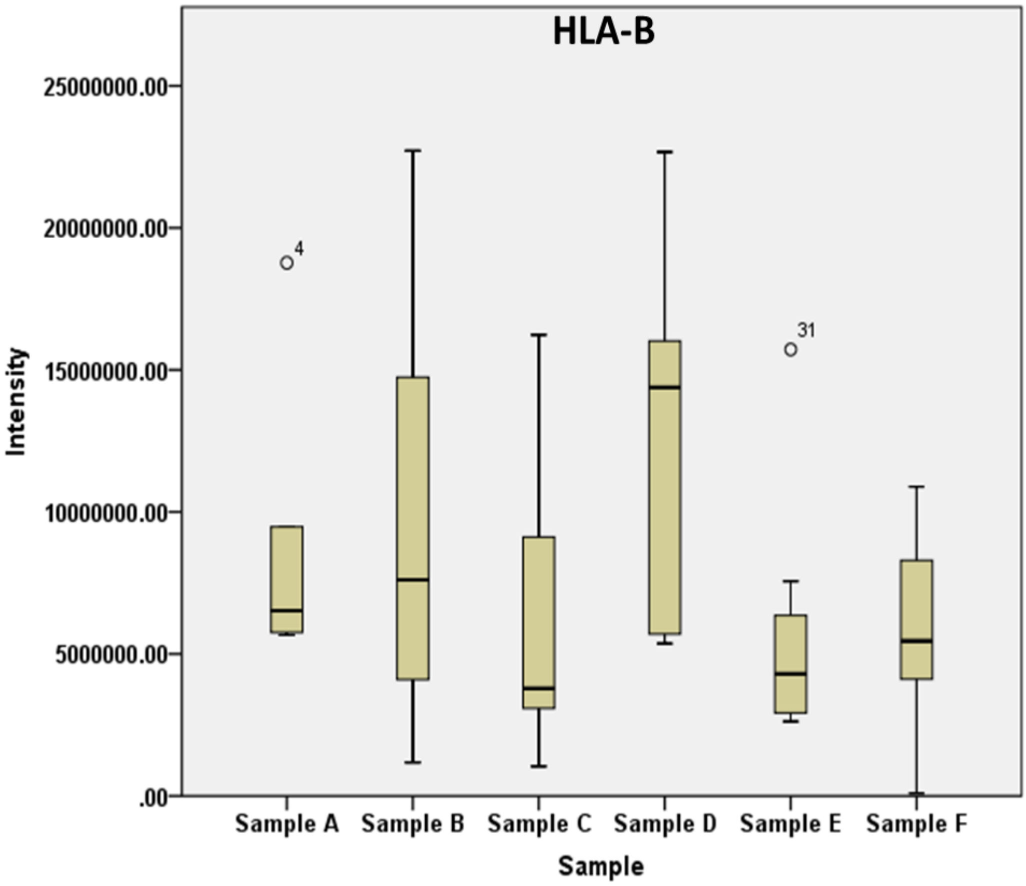
HLA-B
25000000.00
20000000.00
15000000.00
10000000.00
5000000.00
.00
Intensity
4
31
Sample A
Sample B
Sample C
Sample D
Sample E
Sample F
Sample

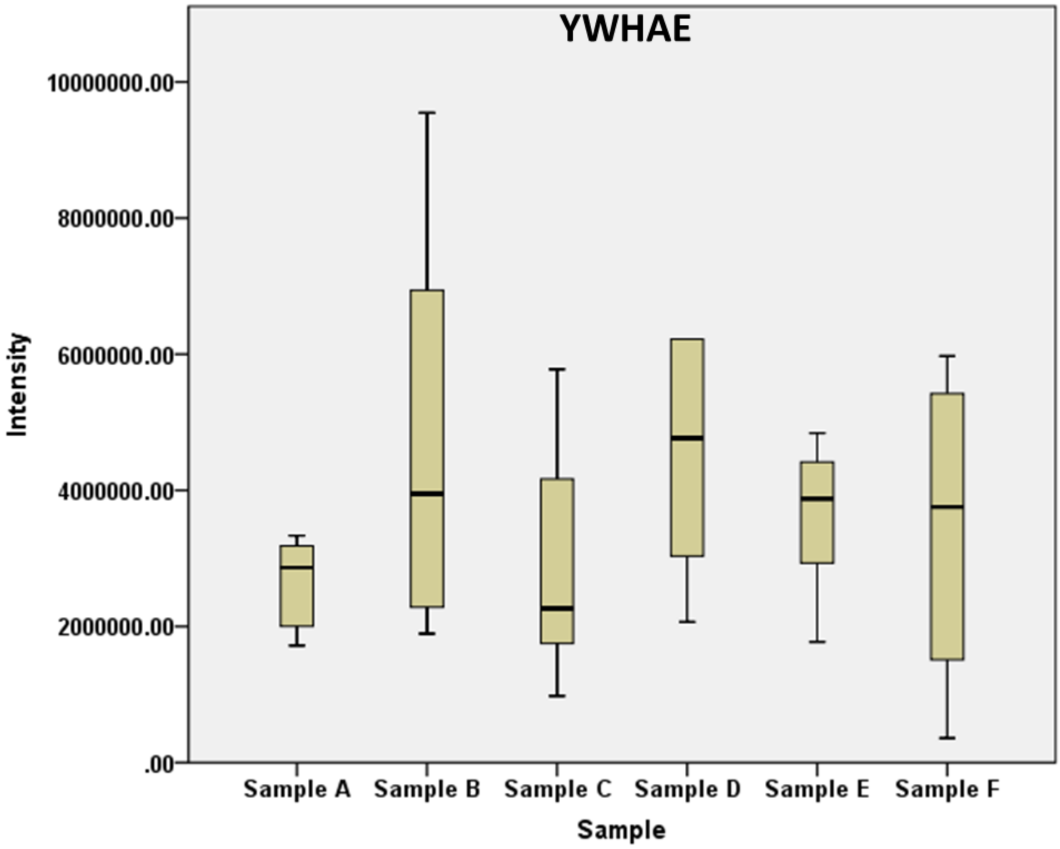
YWHAE
10000000.00
8000000.00
6000000.00
4000000.00
2000000.00
.00
Intensity
Sample A
Sample B
Sample C
Sample D
Sample E
Sample F
Sample

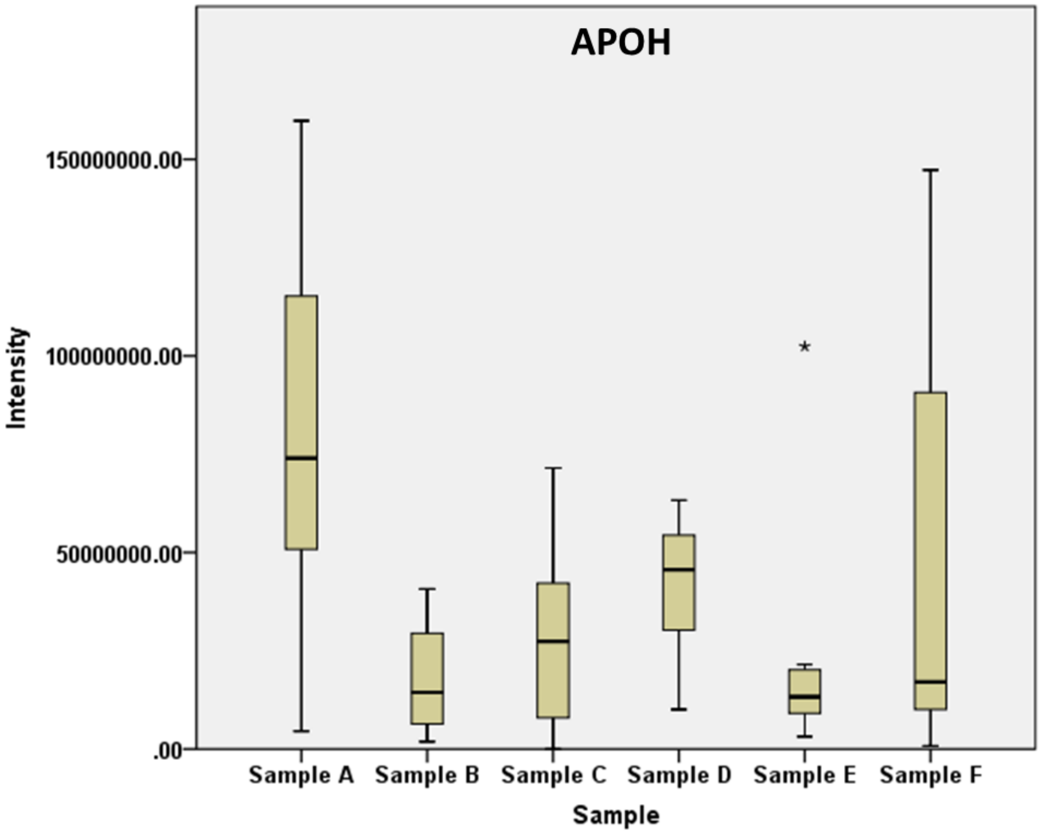
APOH
Intensity
150000000.00
100000000.00
50000000.00
.00
Sample A
Sample B
Sample C
Sample D
Sample E
Sample F
Sample

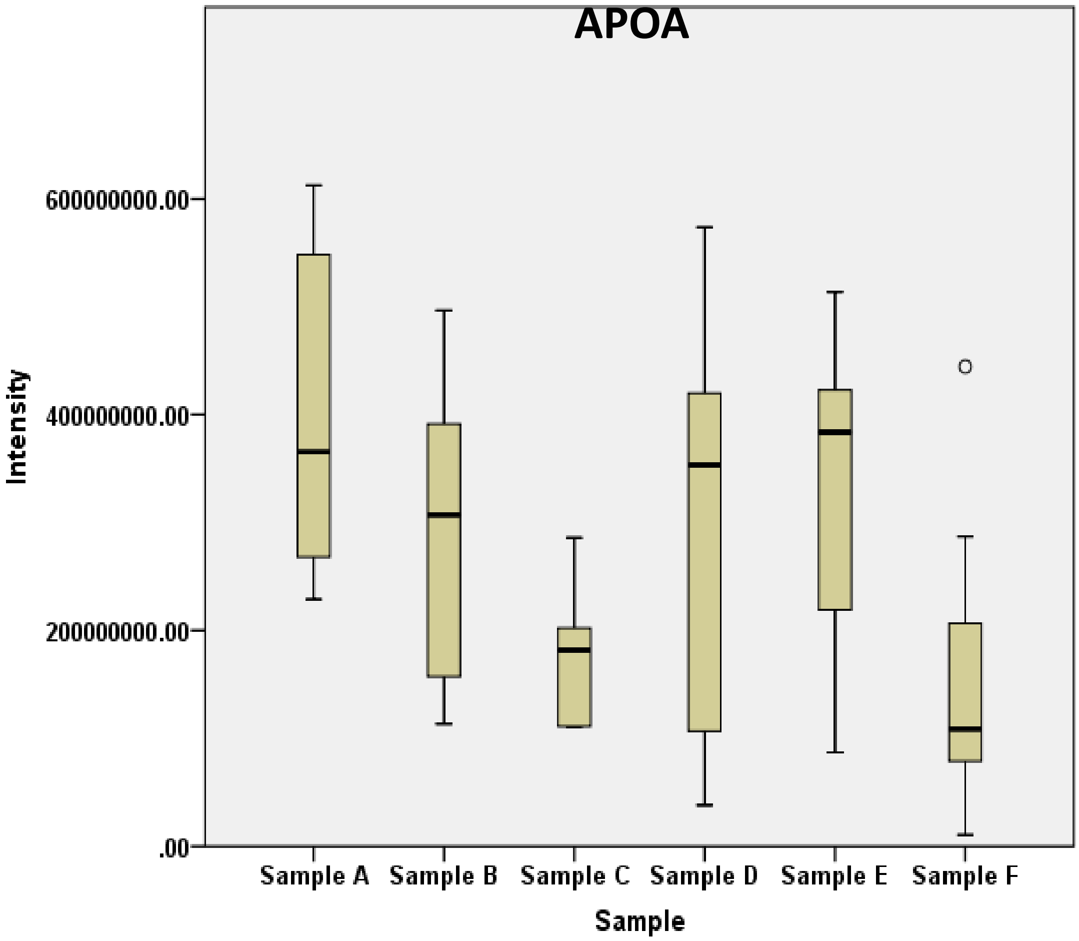
APOA
Intensity
600000000.00
400000000.00
200000000.00
.00
Sample A
Sample B
Sample C
Sample D
Sample E
Sample F
Sample

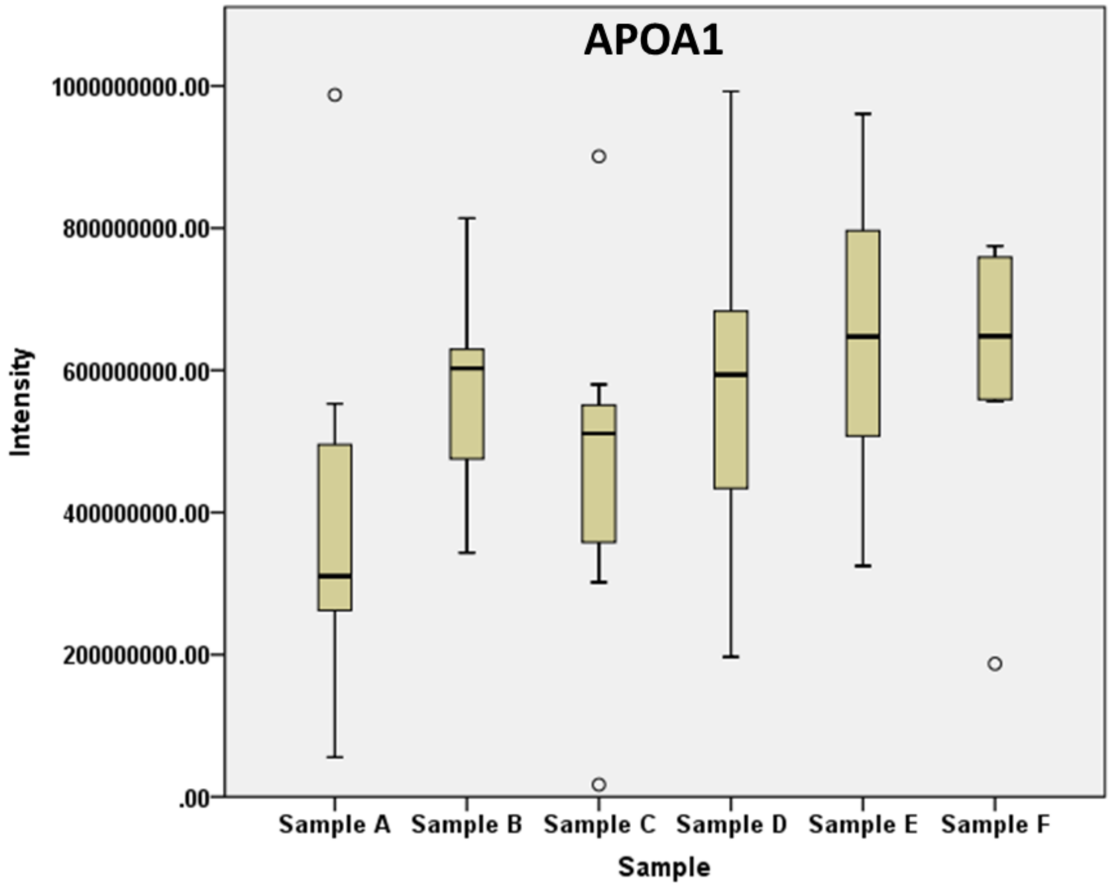
APOA1
1000000000.00
800000000.00
600000000.00
400000000.00
200000000.00
.00
Intensity
Sample A
Sample B
Sample C
Sample D
Sample E
Sample F
Sample

Supplementary Material S2

Complement and Coagulation Cascade KEGG´s pathway

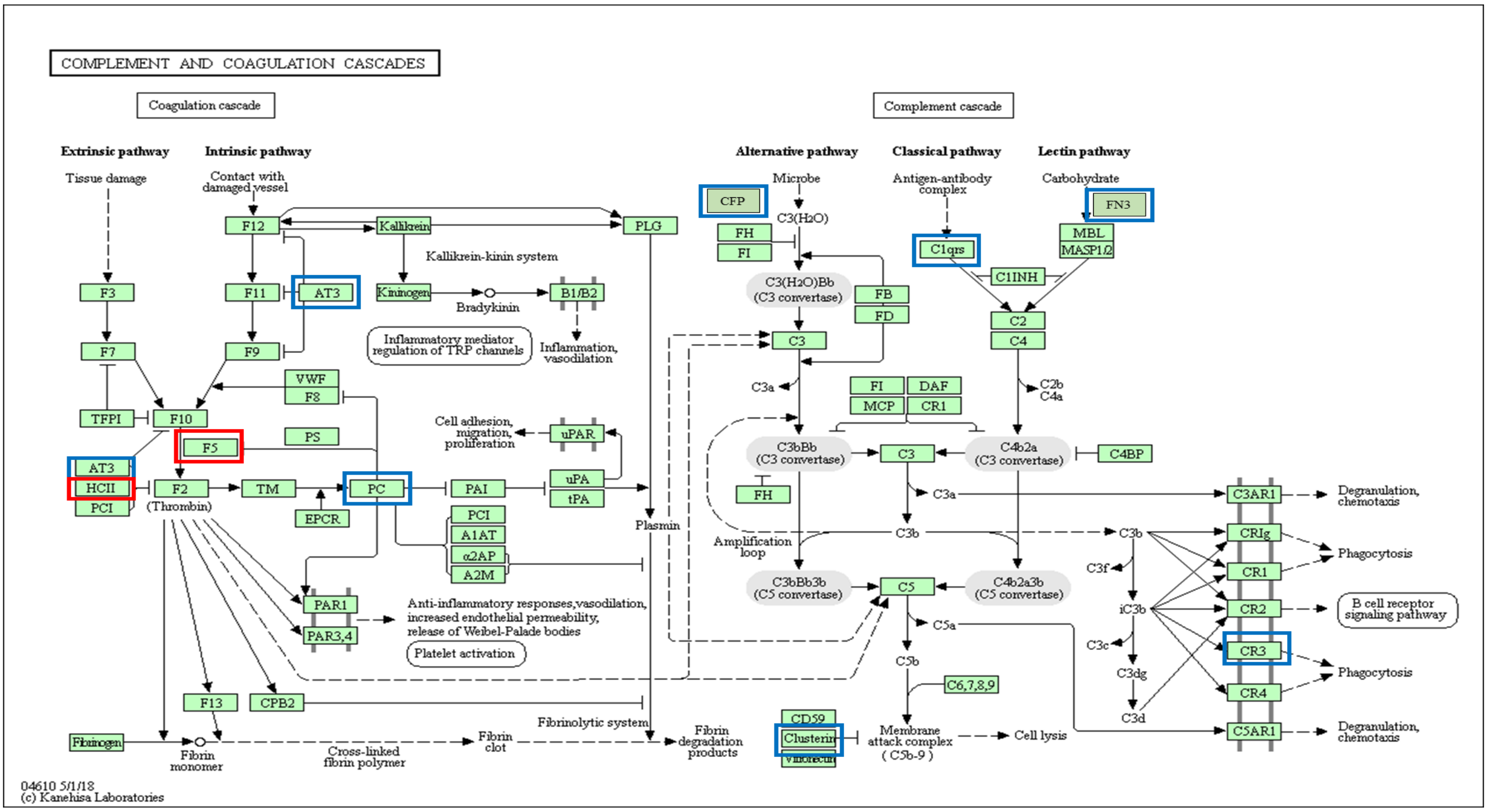

Supplement: Supplementary file 1 [file bsr20182319_Supp1.pdf]
